# Investigation of Betaine and Vaccine Efficacy for Coccidiosis Prevention in Broilers

**DOI:** 10.1007/s11686-024-00967-z

**Published:** 2025-01-24

**Authors:** Asude Gulce Oryasin, Hasan Eren

**Affiliations:** Department of Parasitology, Faculty of Veterinary Medicine, Aydin Adnan Menderes University, Aydin, Turkey

**Keywords:** Betaine, Broiler, Coccidiosis, *Eimeria*, Herbal extracts, Monensin, Vaccination

## Abstract

**Purpose:**

This study aimed to assess the anticoccidial effects of betaine and a vaccine compared to monensin sodium in experimentally induced coccidiosis in broiler chickens.

**Methods:**

600 day-old broiler chickens (Ross 308) were randomly assigned to five groups, each with four replicates of 30 birds. While the control group received a basal diet, two experimental groups received basal diet supplemented with either 100 mg/kg monensin sodium or 2.0 g/kg betaine. The remaining experimental groups received a commercial coccidiosis vaccine alone or in combination with betaine. All chickens were challenged with sporulated field-mixed *Eimeria* species at 20 days of age.

**Results:**

Throughout the study, vaccinated birds showed superior performance in terms of body weight gain (BWG) and feed conversion ratio (FCR) compared to other anticoccidial treatments (*P* < 0.05), followed by betaine, vaccine + betaine, and monensin treatments in descending order. While all anticoccidial regimens significantly reduced fecal oocyst output only at the beginning of the observation period, this effect diminished thereafter (*P* < 0.05). Supplementation with monensin and vaccination against coccidiosis significantly decreased small intestine weight compared to untreated controls (*P* < 0.01), with a numerical decrease observed in chicks fed betaine-supplemented diets and treated with the combination of betaine and vaccine.

**Conclusion:**

Notably, there has been no prior study comparing betaine with monensin sodium and a coccidiosis vaccine. These findings suggest that dietary betaine supplementation and a commercial coccidiosis vaccine containing mixed *Eimeria* spp. may offer benefits in controlling coccidiosis, presenting viable, cost-effective, sustainable, and safe alternatives to conventional ionophore anticoccidials, with added benefits of no residue and reduced resistance hazards for both animal and human consumption.

**Supplementary Information:**

The online version contains supplementary material available at 10.1007/s11686-024-00967-z.

## Introduction

In the broiler farming, one of the most significant and prevalent diseases, particularly affecting the young ones, is considered to be coccidiosis, caused by various species of *Eimeria* within the Apicomplexa phylum. Besides deteriorating the health and condition of all domestic animals, it leads to reduced feed consumption, performance decline, dehydration, body weight loss, and eventually high mortality rates [1–4]. From an economic perspective, the coccidiosis cases in broiler chicken farming result in economic losses exceeding 7–13 billion British pounds [5, 6]. Additionally, coccidial infections can lead to secondary infections such as necrotic enteritis. Among the predisposing factors for necrotic enteritis, caused by *Clostridium perfringens*, are known to be *Eimeria* agents, litter quality, and sudden changes in the ration. This indicates a direct relationship between the pathogenicity of *C. perfringens* and *Eimeria* infections in broiler chicken farming [7–10]. To prevent coccidiosis and reduce the economic losses it causes in poultry farming, anticoccidials have been regularly and successfully used for a long time. Ionophore antibiotics are commonly used anticoccidial agents for preventing poultry coccidiosis [11, 12]. Despite the slow development of resistance to ionophore antibiotic monensin, exposure to infection leads to the development of natural immunity in animals, eliminating clinical symptoms, which supports the continued use of monensin [13]. However, the prolonged and indiscriminate use of anticoccidials has led to the development of resistant *Eimeria* species. The detection of ionophore anticoccidial residues in some organs and tissues of broiler chickens has increased consumer sensitivity, creating reservations against anticoccidials [14]. These developments have accelerated the search for alternative, environmentally friendly, sustainable, and economical solutions to ionophore anticoccidials [15–19]. For all these reasons, there is a rapid global search for alternative methods to ionophore anticoccidials to protect against coccidiosis. Two of the prominent alternative methods so far are vaccines and herbal preparations. Commercial vaccines are available today, and vaccine development studies are ongoing [12]. Coccidiosis vaccines are rotated with anticoccidials during specific breeding periods throughout the year to minimize resistance formation [20, 21]. Due to emerging resistance, current studies suggest the use of natural-origin feed additives defined as herbal extracts [14, 22, 23]. Betaine, an osmoprotectant obtained from sugar beets (*Beta vulgaris*), synthesized by many plants and organisms in nature, has positive effects on poultry growth and performance. In a study where betaine was used alone or in combination with salinomycin, it was reported to reduce mortality in both cases and have a positive effect on body weight gain (BWG) and feed conversion ratio (FCR) [24–26]. Besides different effects on performance and carcass characteristics, betaine has osmoregulatory, anticoccidial, and immunomodulatory activities. Betaine has been reported to inhibit the different developmental stages of *Eimeria* species, reducing the harmful effects of coccidiosis on broiler chickens and improving intestinal structure and function [27]. In conclusion, although clinical symptoms are suppressed with anticoccidial applications, subclinical coccidiosis cases continue intensively, causing productivity losses in animals. Additionally, reasons such as resistance formation to the drugs, increasing costs in developing new anticoccidials, revealing the negative effects of the drugs on human health, and the growing consumer pressure have led to new global searches for alternative, natural, and eco-friendly methods in disease control [28]. A comparative study directly contrasting betaine, monensin and vaccination has not yet been conducted. This study aims to develop an alternative, reliable, and efficient control strategies for the issue awaiting solutions in veterinary medicine and public health.

## Materials and Methods

### Animal Experimentation

A total of 600 broiler chicks (Ross 308) were used in the trial. Immediately after hatching, all chicks in the trial were vaccinated with an inactivated vaccine against infectious bursal and newcastle diseases (AviPro 202 ND-IBD). After weighing the chicks, they were randomly assigned to experimental units in wood shavings-covered compartments, with 30 chicks in each compartment (15 males + 15 females) according to a completely randomized design, resulting in 4 replications and 5 equal groups. The trial management and physical structures were identical for all compartments within a single roof, separated by a service room to prevent cross-contamination of caretakers, equipment, and entry-exit points. The compartments were fully isolated from each other through the service room.

### Feed Materials

The basal diet used in the trial were prepared in powdered form, primarily based on corn-soybean meal. The chicks in the trial were fed broiler starter feed from days 1 to 14, broiler grower feed from days 15 to 28, and broiler finisher feed from days 29 to 48. The feeds were formulated to be isocaloric and isonitrogenous, considering the recommended levels for the hybrid used in determining the nutrient content of the feeds, and they did not fall below the limits specified in the NRC (1994).

### Anticoccidial Selection

A monovalent ionophore anticoccidial compound commonly used in broiler chicken feeds worldwide and in Turkey was selected for the study (COXIDIN^®^ 200 microGranulate, Huvepharma, Sofia, Bulgaria). The recommended minimum and maximum practical application doses for monensin in broiler chickens in European Union countries are 100 to 125 mg/kg of feed. In this study, 100 g of the preparation was added to one ton of feed to achieve the recommended lower limit dose (100 mg/kg).

### Selection of Anticoccidial Effective Plant Extract

When selecting the anticoccidial effective plant extract, attention was paid to using a medicinal plant derived from sugar beets, which is grown sustainably in Turkey. Betaine, whose anticoccidial efficacy has been demonstrated in previous studies, was utilized for this purpose. The commercial preparation of betaine (Betaine HCl) was obtained from Agrana^®^ Ltd. Co. (China).

### Selection of Coccidiosis Vaccine

Chicks were vaccinated against coccidiosis by mixing Livacox^®^ Q commercial vaccine into their drinking water. All commercially available vaccines in the market contain similar *Eimeria* species to the one used in this study. The commercial vaccine used in the trial (Livacox Q^®^, Biopharm, Czech Republic) contained 30,000–50,000 oocysts of attenuated strains of *E. tenella*,* E. acervulina*, and *E. maxima*, and 10,000 oocysts of *E. necatrix* per 1 ml, in a 1% w/v aqueous solution of chloramine.

### Arrangement of the Trial

The trial consisted of 4 replications, each comprising 5 groups with 30 chicks per group. The experimental units were formed by assigning 120 chicks to each group. The arrangement of the trial is shown in Table 1. While the control group received basal diet, the second group received basal diet with the addition of 100 mg/kg monensin sodium. Chicks in the third and fifth groups were orally vaccinated using the drinking water method. For this purpose, vaccination was administered to each compartment through hanging chick drinkers at a rate of 300 ml per compartment. The vaccine solution was dissolved in chlorine-free water at room temperature, mixed well, and then administered at a rate of 10 ml per chick for 4 h. Subsequently, the chicks were transitioned to tap water. Immediately after vaccination, vitamin supplementation was provided (Teknovit, AD3E Plus). The 1st, 2nd, and 4th groups, where no vaccination was applied, were provided with a 1% saline solution equivalent to the volume of the vaccine solution. The chicks in the 4th group were given 2.0 g/kg betaine in addition to basal diet. The chicks in the 5th group were given 2.0 g/kg betaine in addition to both the vaccine and basal diet.

**Table 1:** The arrangement of the trial

1. group	Control	No anticoccidial
2. group	monensin	100 mg/kg monensin sodium of diet
3. group	vaccine	Livacox Q
4. group	betaine	2.0 g/kg betaine of diet
5. group	betaine + vaccine	Livacox Q + 2.0 g/kg betaine of diet

### Animal Husbandry

Chicks were provided with feed and water ad libitum, and lighting was set to 16 h light/8 hours dark. The chicks were housed at a density of 15 chicks/m² in the trial compartments measuring 1.50 × 2.20 m. Each compartment was equipped with 2 hanging chicken feeders, 1 hanging drinker, and 1 electric heater. The temperature in the poultry house, initially set at 33 °C, gradually decreased to 23 °C by the 21st day and was then maintained at 22 °C. Dry wood shavings were spread 6 cm deep on the poultry house floor. Separate equipment and caretakers were assigned for each compartment. The trial was planned in a completely randomized design with random parcels, forming 4 replications and 5 groups, each consisting of 30 chicks in each replication.

### Growth Performance Measurements

Chicks were provided with feed and water ad libitum, and lighting was set to 16 h light/8 hours dark. The chicks were housed at a density of 15 chicks/m² in the trial compartments measuring 1.50 × 2.20 m. Each compartment was equipped with 2 hanging chicken feeders, 1 hanging drinker, and 1 electric heater. The temperature in the poultry house, initially set at 33 °C, gradually decreased to 23 °C by the 21st day and was then maintained at 22 °C. Dry wood shavings were spread 6 cm deep on the poultry house floor. Separate equipment and caretakers were assigned for each compartment. The trial was planned in a completely randomized design with random parcels, forming 4 replications and 5 groups, each consisting of 30 chicks in each replication.

### Experimental Coccidiosis Inoculation and Material Collection

The inoculum obtained from Ankara University, Faculty of Veterinary Medicine, Parasitology Department, containing pathogenic *Eimeria* species (*E. acervulina*,* E. maxima*,* E. tenella*,* E. praecox*,* E. brunetti*,* E. mitis*) determined by Nested PCR, was passaged in 15 chicks at 7 days of age. After treatment with potassium dichromate, sporulated oocysts were obtained from collected feces. An inoculum containing 2 ml of sporulated oocysts (35 × 10^4^ oocysts/ml) was orally administered to all chicks at 20 days of age using a probe. Starting from one week after inoculation, all trial groups were monitored until the end of the study. Clinical checks of animals in the trial groups, fecal examinations, and post-mortem examinations of the deceased animals were conducted daily. Samples of at least 100 g each were collected daily from the feces deposited over a 24-hour period, filled into plastic bags, and stored in a refrigerator at (+ 2–4 °C) until oocyst counts were performed. The homogenized fecal samples, diluted 1/10 (w/v) with tap water, were further diluted 1/10 (v/v) with a saturated saline solution. Oocyst counts were determined using a McMaster slide, expressing the oocyst count in 1 g of feces [29]. The parasitological monitoring of the study was conducted by the Parasitology Department of Adnan Menderes University Faculty of Veterinary Medicine.

### Intestinal Lengths and Organ Weights

On the 26th and 48th days of the trial, 3 broilers from each replication (12 per experimental group) were slaughtered. The weights of the spleen, liver, and small intestine; lengths of intestinal segments (duodenum, jejunum, ileum, cecum); and total intestinal lengths were collected.

### Lesion Score

On the 6th day (26th day) after infection initiation, coccidial lesion formations were scored in all slaughtered chickens. Lesions were examined in terms of duodenum, jejunum, ileum, and cecum. The scoring system developed by Johnson and Reid was used for this purpose [30, 31].

### Histopathological Examination

On the 6th day (26th day) after infection initiation, a section of the jejunum belonging to the control group was stained with hematoxylin-eosin and examined by the Pathology Department of Adnan Menderes University Faculty of Veterinary Medicine.

### Statistical Analysis

All data were analyzed using Statistical Analysis System User’s Guide (SAS) [32]. Data were subjected to ANOVA by using the General Linear Model (GLM) procedure. Each replication constituted an experimental unit. The DUNCAN multiple comparison test was used to assess differences between groups. Since the oocyst yields were not distributed normally, the Kruskal-Wallis non-parametric analysis was employed [32]. Lesion scores were not subjected to statistical evaluation since they frequently contained the value “0 (zero)”. Values expressed as percentages were subjected to arcsine transformation before evaluation. Probability values smaller than 0.05 were considered significant (*P* < 0.05), while larger values were considered insignificant (*P* > 0.05).

## Results

### Growth Performance

The growth performance measurements for the pre-infection period (0–20 days), post-infection periods (21–35, 36–48, and 21–48 days), and the entire 1–48 days are provided in Table 2. No statistically significant differences were observed among the trial groups in terms of performance data during the pre-infection period (0–20 days) (*P* > 0.05). Monensin showed numerical superiority in feed intake (FI), betaine in body weight gain (BWG), and betaine + vaccine in feed conversion ratio (FCR) compared to other groups. After infection (21–35 days), although no statistical significance was found among groups in terms of FI and FCR parameters, the vaccine-applied group demonstrated statistical superiority in BWG compared to other trial groups (*P* < 0.05). Between days 36–48, BWG was significantly higher in the vaccine, betaine, and betaine + vaccine groups than in the control and monensin groups (*P* < 0.05). The best FCR value was observed in the vaccine group, followed by betaine (*P* < 0.05). Similarly, during the post-infection period (21–48 days), although there was no statistical significance among groups in terms of FI, the best results in terms of BWG and FCR were obtained from the vaccine application. Betaine and betaine + vaccine applications also exhibited better FCR compared to the control during the same period (*P* < 0.05). Throughout the entire period (1–48 days), in terms of performance values, the vaccine-applied group maintained its superiority over others, followed by betaine, betaine + vaccine, and monensin (*P* < 0.05).

**Table 2:** Growth performance measurements. Feed intake (FI; g), body weight gain (BWG; g), feed conversation ratio (FCR; g feed/g gain), after broilers were infected with an inoculum containing 35 × 10^4^ oocysts of *Eimeria* spp. at 20 d of age and were provided with diets supplemented with monensin, betaine and vaccine either alone or in combination. Body weight per trial is 45.37 g a, b,c, d,e: values within a column not sharing the same superscript are different at *P* < 0.05

	day 0 to 20			day 21 to 35			day 36 to 48			day 21 to 48			day 1 to 48		
Experimental Groups	FI (g)	BWG (g)	FCR	FI (g)	BWG (g)	FCR	FI (g)	BWG (g)	FCR	FI (g)	BWG (g)	FCR	FI (g)	BWG (g)	FCR
control	860.12	592.13	1.45	1606.50	829.77^b^	1.93	1657.05	862.30^b^	1.92^a^	3532	1692.05^c^	2.08^a^	4392	2284^e^	1.92^a^
monensin	890.65	626.63	1.41	1652.22	868.04^b^	1.90	1593.25	866.09^b^	1.82^abc^	3332	1744^c^	1.91^ab^	4222	2373^d^	1.77^b^
vaccine	843.10	584.63	1.44	1690.75	922.99^a^	1.83	1718.85	1009.91^a^	1.70^c^	3409	1932^a^	1.76^b^	4252	2517^a^	1.68^b^
betaine	886.87	632.13	1.40	1643.17	780.21^b^	1.88	1715.03	967.03^a^	1.77^bc^	3416	1838^b^	1.86^b^	4303	2470^b^	1.74^b^
betaine + vaccine	876.85	584.63	1.50	1663.69	869.36^b^	1.91	1755.58	951.91^a^	1.84^ab^	3419	1821^b^	1.87^b^	4296	2406^c^	1.78^b^
Pooled SEM	19.15	15.51	0.04	43.95	17.15	0.05	40.97	21.50	0.04	80.03	17.78	0.05	80.45	6.56	0.033
*P* value	0.4062	0.0935	0.6794	0.7434	0.0264	0.6627	0.0951	0.0009	0.0431	0.5457	0.0001	0.0224	0.6429	0.0001	0.028

### Oocyst Shedding

Daily fecal samples were collected during the 11 days following infection. The oocyst counts determined in the feces are presented in Table 3. In the initial control after infection (day 6), the groups with added anticoccidial feed additives had lower oocyst counts than the control. However, significant differences were found among groups in terms of both statistical (*P* < 0.05) and numerical differences in oocyst shedding in the following days. Oocyst shedding exhibited periodical variations.

**Table 3:** Daily (as measured at 7 to 18 days post infection) fecal oocyst output in chicks given diet supplemented with anticoccidial after broilers were infected with an inoculum containing 35 × 10^4^ oocysts of *Eimeria* at 20 d of age. a, b,c: values within a column not sharing the same superscript are different at *P* < 0.05 oocyst excretion (unit/g feces)

	day 1	day 2	day 3	day 4	day 5	day 6	day 7	day 8	day 9	day 10	day 11
control	39,150^a^	7412	1400	825	775^b^	17,450	6100	12,875	20,675	435^bc^	575
monensin	10,600^b^	5500	375	400	7000^a^	8000	5950	14,100	14,750	175^c^	225
vaccine	22,000^ab^	5125	1625	750	2075^b^	14,425	6625	17,050	4250	1200^bc^	1900
betaine	27,225^ab^	7450	2225	3550	2300^b^	10,450	6375	28,275	32,425	3625^ab^	1450
betain + vaccine	8700^b^	4775	2425	450	3150^b^	14,975	3850	10,500	9800	6375^a^	1075
Pooled SEM	6661	2393	855	1184	1022	5419	1546	5965	8760	1100	509
*P* value	0.0319	0.8811	0.4925	0.3287	0.0071	0.7451	0.7261	0.2936	0.2444	0.0054	0.1976

### Small Intestine Length and Organ Weights

The impact of coccidial infection on the length of the small intestine and proportional weights of internal organs is presented in Table 4. The weights of the spleen, liver, pancreas, and cecum are expressed as a percentage of the live weight of broiler chickens. No statistical significance was found among groups in terms of spleen and liver weights. There was statistical significance in terms of proportional weight of the small intestine (*P* < 0.05), where the addition of monensin to feed and vaccine application significantly reduced the small intestine weight compared to the control (*P* < 0.01), while betaine and betaine + vaccine applications provided a statistically significant reduction. No significant differences were found among treatments in terms of the total length of the intestine and the lengths of duodenum, jejunum, and ileum (*P* > 0.05).

**Table 4:** The effects of dietary anticoccidial strategies with monensin, betaine and vaccine on the intestinal morphology of broilers exposed to coccidial challenge at 20 d of age. Intestinal lengths and organ weights. ^a, b,c^:values within a column not sharing the same superscript are different at *P*<0.05 BW: body weight

	Spleen % of BW	Liver % of BW	Small intestine weight % of BW	Cecum (cm)	Duodenum %100 g	Jejenum %100 g	Ileum %100 g	Total intestinal length
control	0.09	2.32	4.91^a^	30.08	16.36	40.31	44.26	176.33
monensin	0.09	2.36	4.29^ab^	27.83	15.59	40.62	43.75	182.33
vaccine	0.09	2.38	4.28^ab^	27.50	15.24	40.50	44.25	179.16
betaine	0.09	2.35	3.54^bc^	28.00	14.61	40.12	44.86	181.41
betaine + vaccine	0.09	2.23	2.88^c^	29.66	15.68	40.67	44.63	176.08
Pooloed SEM	0.006	0.005	0.28	1.07	0.54	0.63	0.59	4.39
*P* value	0.9627	0.3277	0.0001	0.3231	0.2530	0.9699	0.6049	0.7930

### Lesion Scores

After 6 days from the onset of infection (26 days old), lesions caused by coccidiosis in different segments of the intestines of a total of 60 chicks (12 from each trial group) were provided in Table 5. Since lesion scores mostly included “0 (zero),” they were not subjected to statistical analysis.

**Table 5:** Lesion scores. SEM DEĞERİ EKLENECEK!!!!!Birden fazla 0 değeri içerdiği için yazılmamıştır

	Number of samples and lesion scores											
control	1.	2.	3.	4.	5.	6.	7.	8.	9.	10.	11.	12.
duodenum	0	0	1.5	0	1	0.5	0	0.5	0.5	0.5	0.5	0
jejenum	0	0	0.5	0	1	0.5	0.5	1	1	0.5	0.5	0
ileum	0	0	0	0.5	0	0.5	0	0.5	0	0.5	0	0
cecum	0	0	0	0.5	0	0	0	0	0	0	0	0
**monensin**	**1.**	**2.**	**3.**	**4.**	**5.**	**6.**	**7.**	**8.**	**9.**	**10.**	**11.**	**12.**
duodeum	0.5	0	1	0.5	0	0.5	1	0.5	1	0.5	0.5	1
jejenum	1	0.5	1	0	0.5	0.5	0.5	1	0.5	0	0.5	0.5
ileum	0.5	0	1	0	0.5	0	0	0.5	0.5	0	0.5	0
cecum	0	0	0	0	0	0	0	0	0	0	0	0
**vaccine**	**1.**	**2.**	**3.**	**4.**	**5.**	**6.**	**7.**	**8.**	**9.**	**10.**	**11.**	**12.**
duodenum	0.5	0.5	0.5	1	0.5	0.5	2	1	1	2	3	0.5
jejenum	0	0.5	0	0.5	0.5	1	2	0.5	1	2	2	0
ileum	0	0.5	0	0.5	0	0.5	2	0.5	1	0.5	1	0
cecum	1	0	0	0	0	0	0.5	0	0	0	0.5	0
**betaine**	**1.**	**2.**	**3.**	**4.**	**5.**	**6.**	**7.**	**8.**	**9.**	**10.**	**11.**	**12.**
duodenum	0.5	2	1	2	2	0.5	1	2	0.5	0.5	1	1
jejenum	0.5	3	0.5	1	1	0	2	1	0.5	0.5	0.5	1
ileum	0.5	0.5	1	0.5	1	0.5	0	0.5	0.5	0.5	0	0
cecum	0	1	0	0	0	0	0	0	0	0	0	0
**betaine + vaccine**	**1.**	**2.**	**3.**	**4.**	**5.**	**6.**	**7.**	**8.**	**9.**	**10.**	**11.**	**12.**
duodenum	0.5	1	1	0.5	0.5	0.5	0.5	0.5	1	0.5	1	0.5
jejenum	0.5	1	0.5	0	0.5	0.5	0	0	0.5	0.5	0.5	0
ileum	0	0	0	0	0.5	0.5	0.5	0	0	0.5	0	0.5
cecum	0	0	0	0	0	0	0	0	0	0	0	0

### Histopathology

**Fig. 1:**
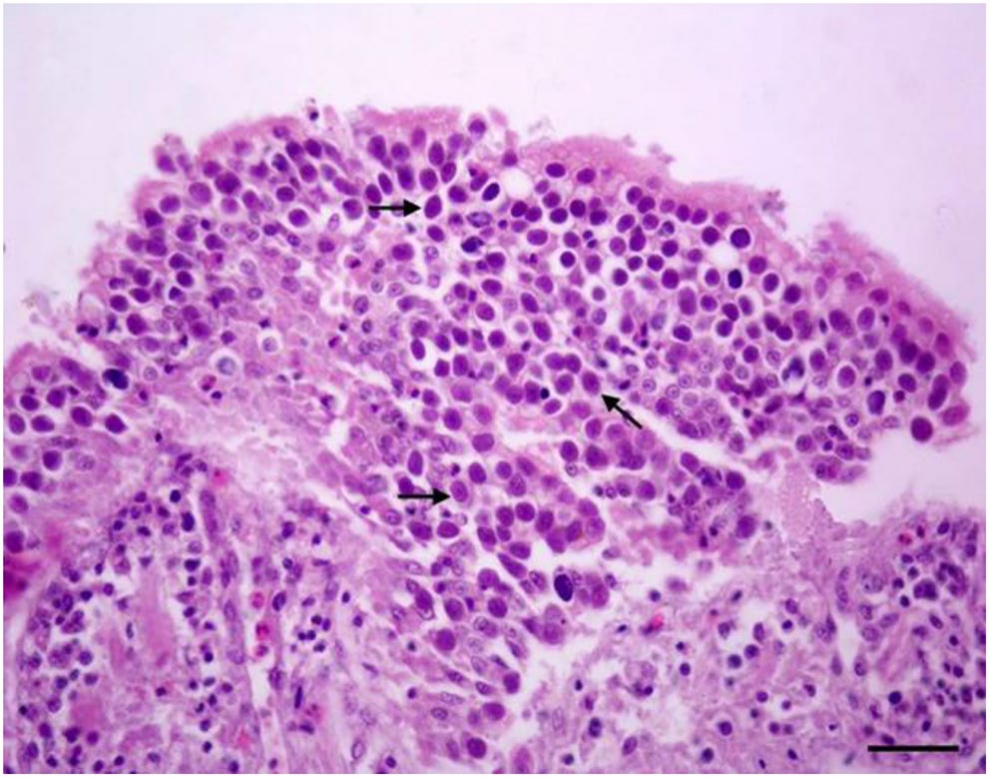
Intraepithelial macrogametes (arrows) in the jejunum. H&E stain. Bar: 30 μm

On the 6th day after infection initiation (26th day), numerous *Eimeria* spp. agents and epithelial tissue shedding were observed in the jejunal section of the control group, as depicted in Fig. 1.

## Discussion

Coccidiosis is a significant disease in poultry farming worldwide, causing damage to the intestinal mucosa, malabsorption, maldigestion, dehydration, diarrhea, weight loss, a drop in feed conversion rates, and increased susceptibility to other diseases. It is primarily caused by *Eimeria* species, obligatory intracellular protozoa. The disease poses a major economic threat, particularly in densely populated poultry farms, leading to substantial financial losses. Parasitic diseases like coccidiosis, in contrast to viral and bacterial infections, are noteworthy due to their complex life cycles, lack of dependence on intermediate hosts, inadequate serological methods for diagnosis, resistance to disinfectants, and the limited efficacy of quarantine practices [33]. In broiler farming, coccidiosis control heavily relies on ionophores, chemical anticoccidials, and, to a lesser extent, coccidiosis vaccines. Concerns have arisen over time regarding the development of resistance to ionophore anticoccidials, increasing costs in developing new anticoccidials, and the negative impact of current anticoccidials on human health. Additionally, consumer pressure has led to a global search for alternative, natural, and environmentally friendly methods of disease control [28]. In recent years, there has been a growing interest in alternative approaches to coccidiosis prophylaxis, moving away from traditional methods [15]. The use of plant preparations provides an alternative in many antiparasitic treatments, including coccidiosis [14, 28]. It has been reported that plant bioactives reduce oocyst shedding, positively affect intestinal lesion scores, and improve performance [16, 34]. To date, there is no study comparing betaine with monensin and vaccines in poultry farming. This study aims to address this gap and develop an alternative, sustainable, economical, reliable, and effective control method for the unresolved issue in veterinary medicine. Coccidial infections in poultry farming can manifest as acute or chronic infections. Subclinical infections, which are less severe, do not exhibit significant increases in mortality and clinical symptoms. The subclinical form of infection leads to damage in the intestinal mucosa, reduced digestion and absorption, weight loss, and decreased feed utilization due to protozoa and pathogenic bacteria [12]. The reasons for the adverse effects on performance as a result of coccidial infection are associated with malabsorption, inflammation, and surface absorption area [35, 36]. As expected, the trial results show that coccidiosis adversely affects growth performance and increases lesion scores [16, 37]. This study aims to investigate the effects of betaine and its combined use with a vaccine on coccidiosis.

In this study, betaine demonstrates anticoccidial effects comparable to monensin. Similarly, Matthews et al. reported in their study on broilers infected with *E. acervulina* that betaine could have an anticoccidial effect. The researchers suggested that the improvement in the performance of broilers given betaine might be associated with increased feed absorption in the intestinal lumen. Subsequent studies have also shown that betaine improves the digestibility of essential nutrients, such as amino acids. However, researchers have not observed a synergistic effect when using betaine and monensin together [37]. Conversely, in healthy chickens not infected with coccidiosis, betaine has been reported not to be effective in maintaining intestinal integrity [38]. These results indicate that exploring betaine’s anticoccidial capacity further is worthwhile. In another study, 14-day-old chickens infected with *E. tenella*,* E. maxima*, and *E. acervulina* were treated with different doses of betaine and salinomycin. The addition of betaine resulted in a decrease in lesion scores and an improvement in feed conversion rates, with no mutual interaction observed between betaine and salinomycin [39]. Kettunen et al. measured the jejunal epithelial crypt-villus ratio in broilers infected with *E. maxima* at 21 days of age. They observed that dietary supplementation of betaine reduced the crypt-villus ratio in both coccidia-infected and non-infected chickens. According to the researchers, betaine protects intestinal villi against coccidia infection and stabilizes mucosal structure, supporting the notion that betaine is a significant intestinal osmolyte in broilers [40]. In another study with similar outcomes, Küçükyılmaz et al. investigated the anticoccidial effects of live attenuated coccidiosis vaccine, essential oil blend, and sodium monensin. They found that the coccidiosis vaccine could be as effective as sodium monensin, improving growth performance and reducing the severity of infection. The vaccine induced a temporary decrease in performance post-vaccination, which was quickly resolved [41]. While some researchers reported that betaine has ionophore-supporting effects, and it does not occur when used with ionophore anticoccidials like monensin and narasin [37], other studies have suggested the opposite [42]. Augustine et al. mentioned that the success of betaine-derived anticoccidial varies based on changes in chicken vaccination programs [43]. Coccidial infection significantly increases the length and weight of the cecum, altering intestinal morphology [44]. In this study, the most significant decrease in small intestine weight was observed for betaine + vaccine compared to the control group (*P* < 0.01).

In this study, there is no classical lesions caused by coccidial infection were found in the intestinal epithelium tissue. The transformation in performance and mortality rates supports this observation. The high maternal immunity of chicks and their overall good health or resistance to damage from mixed infections may contribute to this result. As observed in previous studies with low infection severity [45], experimental coccidiosis in this study negatively affected feed conversion ratios and oocyst counts but did not increase mortality significantly. It is suggested that coccidial or bacterial infections are not sufficient to observe a significant increase in mortality. The increase in intestinal length and cecal weight due to coccidial infection reversed to normal dimensions with the addition of betaine, a plant extract to the diet, supporting the results of performance and oocyst count. The findings from this study are considered original and instructive in supporting the use of natural extracts in coccidiosis control. In the group where the vaccine was applied, it was observed that oral vaccination against coccidiosis at 2 days of age could be as effective in coccidiosis control as conventional anticoccidial treatments. Previous similar studies have also shown successful results with the vaccine [41, 46, 47]. However, in this study, combining the vaccine with betaine yielded more positive results than using the vaccine or betaine alone. In conclusion, the findings from this study demonstrated that betaine, a plant extract, exhibited anticoccidial effects comparable to sodium monensin, alleviating the destructive effects of coccidial infection on broilers. Although severe clinical symptoms and a significant increase in mortality were not observed, experimental coccidiosis negatively affected feed conversion ratios and oocyst counts, leading to significant losses in BWG and FCR. The use of coccidial vaccination, alone or combined with betaine, proved to be an effective anticoccidial application. Further studies are needed to understand the anticoccidial mechanisms of betaine against a single *Eimeria* species and under conditions of high infection severity, providing insights into alternative active substances for anticoccidial use. Amerah and Ravindran reported a reduction in duodenum, jejunum, and total lesion scores in broilers experimentally infected with coccidia when supplemented with betaine [48]. Coccidiosis reduces energy and feed digestibility, leading to worsened feed conversion rates. Betaine supplementation minimizes the adverse effects of coccidia, positively influencing feed digestion and utilization. Betaine’s osmotic activity is attributed to its dipolar zwitterionic character and high solubility in water [49]. When cells are exposed to osmotic and ionic stress, betaine acts as an osmoprotective agent by replacing inorganic ions, protecting enzymes and cell membranes from inactivation due to inorganic ion inactivation [50]. The entry of oocysts into the intestinal epithelium represents a condition representing osmotic and ionic stress, causing morphological and physiological changes in the intestinal epithelium. These conditions create an opportunity for betaine to exert its effectiveness, and the positive results obtained in this study regarding betaine can be attributed to the osmotic and ionic stress associated with coccidiosis. Monensin is known to play a role in maintaining the balance of bacterial populations in the intestinal microflora [51]. This improvement in microbial balance has increased the availability of nutrients and the transit rate of feed through the intestines, stimulating the secretion of endogenous digestive enzymes [52]. The decrease in performance due to coccidial infection is associated with a reduction in the absorption surface area, malabsorption, and inflammation [35, 36]. Previous studies have reported that plant extracts exhibit anticoccidial effects and reduce fecal oocyst shedding [14]. The inhibitory effect of monensin on coccidia-induced lesions in the intestines is also a known fact [53]. Similar to the results obtained by some researchers [44], this study also observed a reduction in fecal oocyst shedding with betaine compared to the control group. The stress caused by coccidial infection leading to malabsorption and maldigestion results in a reduction in the absorption surface area of the intestines, negatively affecting intestinal histomorphology [54]. However, it is believed that this condition can be corrected with prophylactic methods such as probiotic and prebiotic preparations [35, 36]. The increase in villus height and width contributes to better digestion and absorption of feed [55]. Subclinical coccidiosis exhibits typical symptoms, as previously indicated in studies [56]. In this experimental infection, minimal mortality was observed, while it adversely affected feed conversion ratios and yield of carcass and edible organs (YEO). The applied anticoccidial control procedures throughout the trial were successful in mitigating the negative impacts of the infection on growth and performance. Preservation of litter quality during the trial is believed to limit the cycle between the parasite in the litter-crop-intestines-fecal matter, resulting in reduced oocyst shedding. In contrast to previous studies showing a linear increase and subsequent gradual decrease in fecal oocyst shedding within ten days after experimental oral oocyst infection [34, 45], our study observed prolonged oocyst shedding after experimental infection. The fluctuating pattern of oocyst shedding in this study is different from the trends observed in previous research, partially explained by McDougald’s hypothesis suggesting that each *Eimeria* species exhibits its oocyst shedding pattern at different times during mixed *Eimeria* spp. infection [56]. Girgis proposed that mixed infection cases could occur in a flock at various times due to cross-immunity deficiency, leading to fluctuating oocyst shedding. Periodic fluctuations in oocyst numbers were observed in fecal samples collected during the days following infection in this study [57]. This pattern is referred to as the “trickle effect,” as described by Joyner [58]. Consequently, whether applied alone or in combination with the vaccine or the vaccine alone, betaine significantly reduced fecal oocyst shedding. Fecal oocyst counting is a primary method for diagnosing coccidiosis, but the relationship between oocyst numbers in feces and performance is not clear. Future studies are needed to explore the mechanisms underlying this relationship. Similar to studies reporting reduced fecal oocyst shedding in experimentally infected broilers treated with plant extracts [59], this study demonstrates that betaine, whether applied alone or in combination with the vaccine or the vaccine alone, significantly decreased fecal oocyst shedding. Vaccination of broilers at 2 days of age negatively affected feed intake and live weight during the pre-infection period (days 0–20), resulting in reduced performance of FCR. This aligns with the findings of Williams, who reported a transient performance decrease in chicks due to coccidiosis vaccination. Although performance was depressed in the initial period, growth was compensated for in the final period. The BWG after vaccination was 10% higher than in the group receiving sodium monensin. Similar improvements were observed in FCR in vaccinated chickens [20]. Based on the observations in this study and some conducted by other researchers, proper coccidial vaccination of broilers appears to significantly reduce oocyst shedding and lesion scores compared to traditional anticoccidials [47]. Betaine, with its osmoprotectant effect, has positive effects on poultry growth and performance. In a study where betaine was used alone or in combination with salinomycin, both conditions reduced mortality, positively affected live weight gain, and improved feed efficiency. Studies in tissue culture have shown that the combination of betaine and salinomycin reduces *E. acervulina* invasion in cells. This combination is assumed to have a direct effect on the development of *Eimeria* pathogens in animals and an indirect effect by supporting intestinal structure and function through its osmoprotectant effect [24]. Virtanen et al. reported a decrease in lesion scores in chicks where betaine was added to the ration along with salinomycin, while Zimmerman et al. stated that betaine had no effect on lesion scores [60, 61]. Betaine, when used in combination with ionophore anticoccidials such as monensin and narasin, has been reported not to affect BWG and FCR [37]. However, other studies have suggested that using betaine with ionophore anticoccidials helps preserve intestinal health [40, 60] and reduce lesion scores [62]. Studies have also shown that betaine enhances broiler performance, potentially amplifying the effects of anticoccidials [63, 64]. Infected chickens with *E. acervulina*, the addition of 500 mg/kg of betaine to the diet has been reported to balance epithelial cell osmolarity in the duodenum, while a dose of 1000 mg/kg increased leukocyte levels in the villus and lamina propria [65]. In a study where betaine was added to the diets of chickens experimentally infected with *Eimeria* species along with ionophore-effective anticoccidials like salinomycin, betaine positively influenced BWG on day 21 but was ineffective on day 45. The addition of betaine to the ration with ionophore anticoccidials can alleviate the adverse effects of ionophores on intestinal water balance. Betaine improves broiler performance directly by enhancing performance and indirectly by preventing invasion and development of coccidiosis in chickens, Augustine et al. suggested [64]. In conclusion, this study demonstrated that betaine possesses anticoccidial activity comparable to monensin sodium, emphasizing its osmoprotectant properties. The combination of the vaccine with betaine did not exhibit synergistic or additive effects compared to their individual applications. The results obtained from this study indicate that betaine and vaccine applications could be sustainable, economical, reliable, and effective alternative control methods for poultry coccidiosis compared to conventional anticoccidials.

In this study, betaine demonstrated anticoccidial effects comparable to sodium monensin, alleviating the destructive impact of coccidial infection on broilers. Although severe clinical symptoms and significant increases in mortality were not observed, experimental subclinical coccidiosis led to considerable losses in FCR and oocyst counts, resulting in performance declines. These findings are original and informative in supporting the use of plant extracts in coccidiosis control. Recent studies further support these findings. For example, Javanmiri et al. compared various anticoccidial interventions, including anticoccidial drugs, probiotics, synbiotics, phytochemicals, and vaccines, in broilers challenged with *Eimeria* spp. Their research underscores the potential of phytochemicals as effective prophylactic options against coccidiosis, especially given the growing issue of resistance to conventional treatments [66]. Similarly, Al-Hoshani et al. demonstrated that star anise essential oil significantly reduced oocyst shedding and lesion scores, showing efficacy comparable to traditional treatments [10].

Additionally, Geng et al. investigated a botanical product based on eucalyptus, apigenin, and eugenol, finding that these natural compounds mitigated the damage from *Eimeria tenella* infection in broilers [67]. This aligns with the anticoccidial effects observed with betaine in this study, further supporting the use of plant bioactives, which may offer sustainable alternatives due to their diverse mechanisms of action. Likewise, Hailat et al. reported effective field results using phytogenic products to control coccidiosis in broilers, mirroring the reduced severity in intestinal lesions and oocyst shedding observed in our research [68].

These recent studies collectively suggest that plant-based strategies, including compounds like betaine, show promising potential for integration into broiler management systems, potentially complementing or even replacing conventional anticoccidials. With further research, these alternatives could contribute to a more sustainable and resilient approach to coccidiosis management in poultry farming.

Vaccination in coccidiosis, compensating for the decrease in oocyst shedding and growth performance, is as effective in reducing infection severity as sodium monensin. Additionally, despite observing a sudden drop in performance, it is a temporary decline. Combining the vaccine with betaine in mixed coccidia infection has shown a similar effect in reducing oocyst shedding when compared to the individual use of betaine and the vaccine. This suggests that combination strategies, particularly under conditions of high pathogenicity from *Eimeria* spp. infection, may be instructive for the long-term control of coccidiosis in broilers. In conclusion, the findings from this study demonstrate that betaine, a plant extract, exhibits anticoccidial effects comparable to sodium monensin, mitigating the destructive effects of coccidial infection on broilers. Although severe clinical symptoms and significant mortality were not observed, experimental subclinical coccidiosis led to substantial losses in carcass yield and edible organ weights. The use of coccidial vaccination alone or in combination with betaine was shown to be an effective anticoccidial practice. Further research is needed to elucidate the anticoccidial mechanism of betaine against a single *Eimeria* species and under conditions of high infection severity. This would facilitate a clearer understanding of alternative substances with anticoccidial efficacy by deciphering their mechanisms of action. While the anticoccidial effects of plant preparations are well-known, comprehensive studies are required to fully elucidate their mechanisms of action.

## Electronic Supplementary Material

Below is the link to the electronic supplementary material.

Supplementary Material 1

## Data Availability

No datasets were generated or analysed during the current study.
